# Overcoming the Limitations of CRISPR-Cas9 Systems in *Saccharomyces cerevisiae*: Off-Target Effects, Epigenome, and Mitochondrial Editing

**DOI:** 10.3390/microorganisms11041040

**Published:** 2023-04-16

**Authors:** Genki Sato, Kouichi Kuroda

**Affiliations:** 1 Division of Applied Life Sciences, Graduate School of Agriculture, Kyoto University, Sakyo-ku, Kyoto 606-8502, Japan; 2 Department of Molecular Chemistry and Engineering, Kyoto Institute of Technology, Sakyo-ku, Kyoto 606-8585, Japan

**Keywords:** *Saccharomyces cerevisiae*, genome editing, CRISPR-Cas9 system, CRISPR Nickase, epigenetics, mitochondrial DNA, DNA/RNA hybrid

## Abstract

Modification of the genome of the yeast *Saccharomyces cerevisiae* has great potential for application in biological research and biotechnological advancements, and the CRISPR-Cas9 system has been increasingly employed for these purposes. The CRISPR-Cas9 system enables the precise and simultaneous modification of any genomic region of the yeast to a desired sequence by altering only a 20-nucleotide sequence within the guide RNA expression constructs. However, the conventional CRISPR-Cas9 system has several limitations. In this review, we describe the methods that were developed to overcome these limitations using yeast cells. We focus on three types of developments: reducing the frequency of unintended editing to both non-target and target sequences in the genome, inducing desired changes in the epigenetic state of the target region, and challenging the expansion of the CRISPR-Cas9 system to edit genomes within intracellular organelles such as mitochondria. These developments using yeast cells to overcome the limitations of the CRISPR-Cas9 system are a key factor driving the advancement of the field of genome editing.

## 1. Introduction

The CRISPR-Cas9 system is a highly effective tool for genome editing due to its ability to precisely modify targeted genomic regions by altering only a 20-nucleotide sequence within the guide RNA (gRNA) [1]. Although the yeast *Saccharomyces cerevisiae* traditionally allows for efficient alteration of its genomic DNA through homologous recombination (HR) by introducing donor DNA with short homology arms [2], the use of the CRISPR-Cas9 system has gained significant attention in yeast since its initial report [3]. In particular, the Cas9/gRNA complex efficiently induces double-strand breaks (DSBs) in vivo, allowing the simultaneous editing of multiple genomic targets through the high homology-directed repair (HDR) activity of yeasts [4]. The CRISPR-Cas9 system has been successfully employed in various state-of-art gene-editing techniques for yeasts, such as the deletion of almost all genes related to a specific cellular process [5], the construction of lengthy metabolic pathways containing more than 30 exogenous genes [6], and the generation of a pool of genome-wide gene knockout, downregulation, and overexpression libraries in a single step [7].

**Figure 1:**
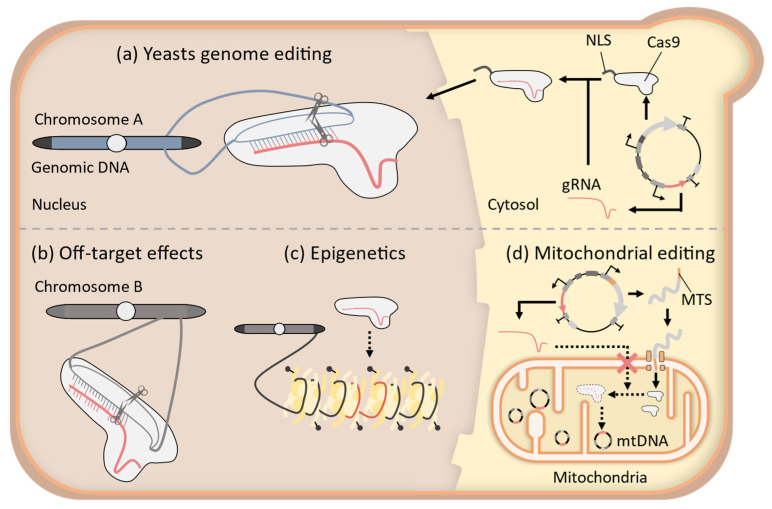
The CRISPR-Cas9 system has several limitations in yeast genome editing. (**a**) Process of common yeast genome editing with CRISPR-Cas9 system. (**b**) gRNA can recruit Cas9 to non-target sequences that are similar to the target sequences. Inducing breaks at these sites causes off-target effects. (**c**) The activity of the Cas9 protein cannot alter the epigenetic state of the target sequence. Moreover, recruitment of the Cas9/gRNA complex can be inhibited by the epigenetic state. (**d**) Cas9 protein can be transported to mitochondria by fusion with a mitochondrial targeting sequence (MTS). However, the transportation mechanism of polynucleotides, such as gRNA, to the mitochondria is currently unclear; therefore, the application of the CRISPR-Cas9 system to mitochondrial DNA (mtDNA) is limited.

However, the CRISPR-Cas9 system, which was originally developed using the Cas9 protein from *Streptococcus pyogenes* Cas9 (SpCas9) and gRNA [1], has some limitations in genome editing (Figure 1). One such limitation is the specificity of target sequences. The gRNA can bind to non-target sequences similar to the intended target sequence and recruit Cas9 protein, leading to DSBs in the non-target region of genomic DNA [8,9]. When DSBs are repaired through non-homologous end-joining (NHEJ), insertions or deletions of nucleotides (indels) can be introduced at the site of the break, and unwanted mutations are thereby caused in the genome. The process is called the off-target effect of the CRISPR-Cas9 system. Off-target effects hinder the construction of strains with the desired genotype [10,11] and can decrease on-target editing efficiency by consuming cellular Cas9/gRNA complexes intended for on-target genome editing [12]. Therefore, a CRISPR-Cas9 system with high precision is necessary for more flexible genome engineering in yeast.

The CRISPR-Cas9 system relies on the use of nucleases, which means that it can only modify genomic DNA sequences and alter gene products. As a result, cellular phenomena beyond genomic sequences are outside the scope of the CRISPR-Cas9 system. A representative example of such a phenomenon is epigenetics, which refers to changes in gene expression caused by a variety of factors independent of the DNA sequence. The well-known examples are chemical modifications of genomic DNA and histone proteins that affect chromatin condensation states and gene expression regulation [13]. Because chemical modifications of DNA and histones are distinct from the DNA nucleotide sequence, these epigenetic modifications cannot be directly altered by the nuclease activity of the CRISPR-Cas9 system. However, epigenetic differences in cells are frequently linked to important eukaryotic cell phenotypes such as human diseases [14]. To advance epigenetic research in eukaryotic cells, it is necessary to develop technology to perform epigenome editing in conjunction with CRISPR-Cas9 tractability in yeast.

Conventional CRISPR-Cas9 systems target DNA in the nucleus of eukaryotic cells. However, potential genome editing targets are also found in some cellular organelles such as mitochondria and chloroplasts. In particular, mitochondria have attracted significant attention as targets for genome editing due to their potential for treating human genetic diseases arising from mutations in mitochondrial DNA (mtDNA) [15]. In recent years, the use of genome editing for the treatment of human genetic diseases has gained increasing interest [16], leading to the development of various genome editing tools for mtDNA editing using the protein-guided recruitment of DNA-editing enzymes to the target sequence [17,18]. However, the use of more flexible RNA-guided recruitment systems such as the CRISPR-Cas9 system is limited because of the challenge of efficient delivery of gRNA to mitochondria, which are isolated from other organelles by two selectively permeable membranes [19,20]. Developing a mitochondrial CRISPR-Cas9 system is a significant challenge for mtDNA editing research because of the need for efficient transport of RNA into the mitochondria [21]. In this area, yeasts have the potential to be a model organism, as suggested by the successful use of RNA motifs derived from yeasts [22] and microprojectile transportation for mitochondrial genome editing [23].

In this review, we provide a brief overview of developments that aim to overcome the limitations of CRISPR-Cas9 system in yeast. Our focus is on the aforementioned challenges for off-target effects, editing of epigenetic information, and editing of mtDNA. These novel systems were developed by exploiting the ease of yeast genetics and the potency of its platform for genome editing tool development. These examples demonstrate that the development of CRISPR-Cas9 systems utilizing yeast cells to overcome these limitations will continue in the future.

## 2. Improvement of CRISPR-Cas9 System for Precise Genome-Wide Editing in Yeast

**Figure 2:**
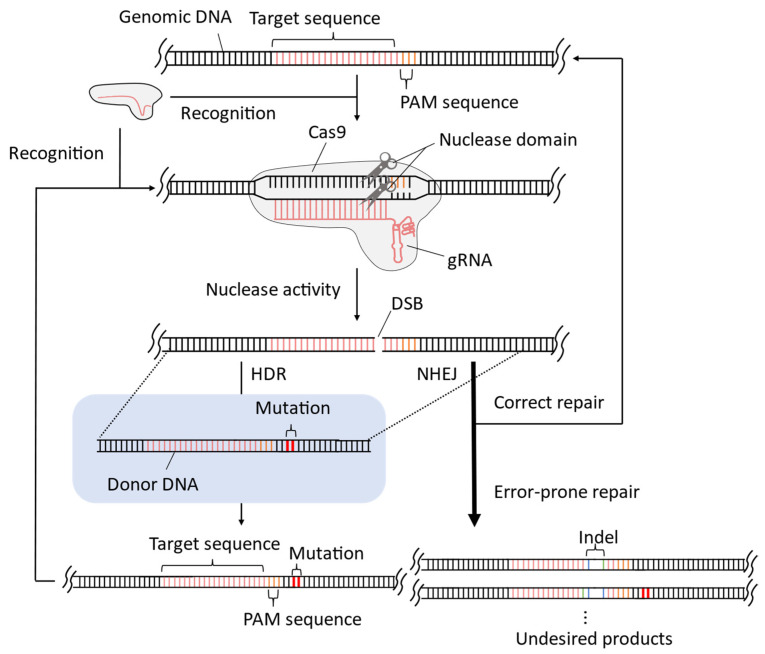
Donor DNA-mediated gene editing may cause unintended mutations at the target site. The introduced DSBs promote the NHEJ pathway, leading to the production of sequences with undesired indels. If donor DNA lacks sufficient nucleotide alterations in its target and PAM sequences, the edited sequence is still recognized and cleaved by Cas9 even when the HDR pathway is induced by donor DNA. As a result, the proportion of edited sequences containing undesired indels in the target and in PAM sequences will increase.

The CRISPR-Cas9 system can sometimes cause unintended sequence alterations at non-target sites, which are referred to as off-target effects. Off-target effects result from DNA breaks and the subsequent DNA repair processes. DNA breaks for off-target effects occur in two steps. First, the gRNA recruits the Cas9/gRNA complex to sequences that are similar, but not identical, to the target sequence. Second, Cas9 nuclease breaks DNA despite the mismatch between the gRNA and genomic DNA (Figure 1). The binding of the Cas9/gRNA complex to the target sequence depends on the 20-base complementarity between the gRNA and genomic sequence. Therefore, gRNA can bind and recruit Cas9 to similar genomic sequences, owing to the degree of tolerance for RNA–DNA hybridization mismatches [9,24]. Although the nuclease activity of Cas9 is enhanced by complementary hybridization and requires the presence of the protospacer adjacent motif (PAM) sequence (Figure 2), the cleavage kinetics of the Cas9 protein allows activity to be elicited depending on the extent and mode of mismatches between gRNA and genomic DNA [8,10,12]. For instance, mismatches located distant from the PAM sequence are less likely to affect nuclease activity [25,26]. Similarly, mismatches that are not contiguous, particularly when spaced more than four bases apart, are less likely to affect nuclease activity [8]. Even in the most critical step of PAM sequence recognition [27], certain PAM-like sequences are permissive for Cas9 nuclease activity [24,28].

Therefore, the potential for nuclease activity at non-target sites of the Cas9/gRNA complex can be minimized through the judicious design of the target sequences of the gRNA, using computational methods to rank the target sequences based on mismatched properties within the whole-genomic sequences of the target cells [29]. In addition, the optimization of Cas9 abundance and activity within cells can also be effective. High concentrations of Cas9/gRNA complexes increase the probability of off-target reactions [12] because of their off-target binding property at a genome-wide level in yeast [30]. However, the most critical factor causing off-target effects is the ability of Cas9 to induce DSBs, which increases the rate of off-target effects by promoting error-prone NHEJ during DNA repair and the introduction of indels [31]. Furthermore, if two DSB ends at different chromosomal loci are connected, global genomic alterations such as chromosomal translocation [32] can be induced.

In addition, DSBs can cause unintended mutations in target sequences, even when the Cas9/gRNA complex is accurately targeted to the desired sequence and HDR is performed using donor DNA [33,34] (Figure 2). Genomic DNA sequences around DSBs are replaced by the donor DNA template in the HDR process, resulting in precise modification of the genomic DNA to the donor DNA sequence. However, in the case of an exceptional donor DNA sequence, such precise genome editing via HDR cannot be performed. The CRISPR-Cas9 system recognizes only the target sequences, which are followed by a specific sequence of a few bases known as the PAM sequence. If the donor DNA lacks sufficient alterations in the target or PAM sequence, the Cas9/gRNA complex can continue recognizing and introducing DSBs into the target sequence even in the case of precise genome editing with HDR. This process can only be halted if NHEJ-mediated repair with an indel occurs in the target sequence or PAM sequence, leading to an increase in unintended mutations in the genome editing outcome. To avoid the risk of unintended mutations during precise genome editing using such donor DNA, only bases located 20 nucleotides upstream or within PAM sequences can be edited. Therefore, only 68.4% of the bases in the genomic DNA of yeast cells can be precisely edited [33].

**Figure 3:**
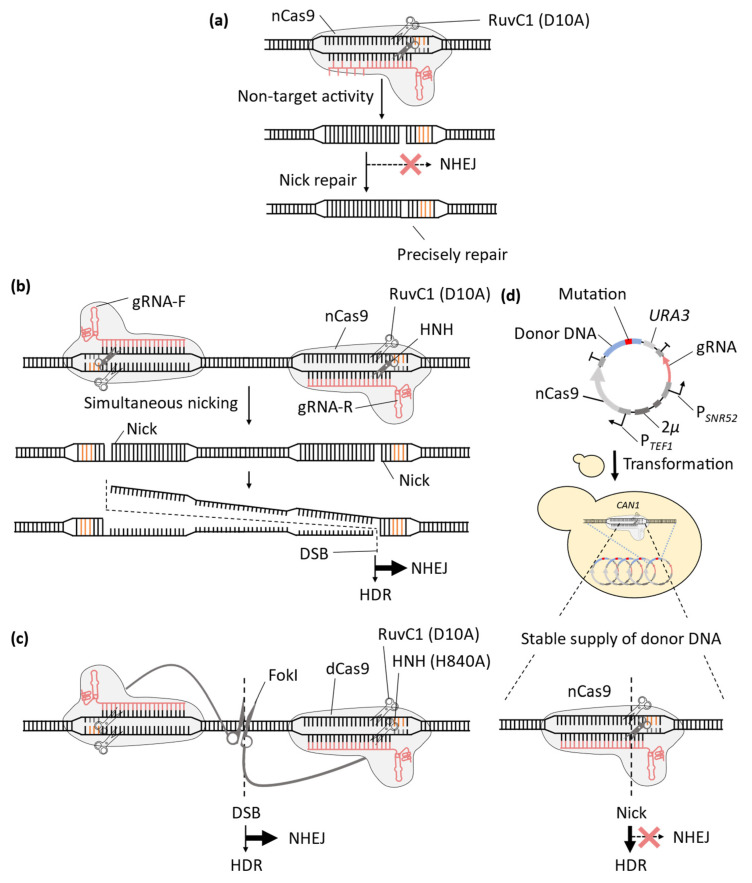
The development of CRISPR-Cas9 systems for reducing off-target effects. (**a**) The nCas9 protein only introduces nicks to the target sequence, preventing NHEJ after the nuclease activity of nCas9 at non-target sequences and thereby decreasing off-target effects in the genome. (**b**) Double-nicking methods use a pair of gRNAs for a single target sequence to induce DSBs only in the region complementary to both gRNAs. (**c**) FokI-dCas9 method does not induce any DNA strand breaks in the region complementary to only one gRNA. (**d**) CRISPR Nickase system, which employs a single gRNA, enables NHEJ-free on-target editing by using a stable supply of donor DNA from multi-copy plasmids.

Therefore, to improve the accuracy of the CRISPR-Cas9 system by minimizing off-target effects, systems that do not generate DSBs are desirable. To this end, researchers developed a modified version of SpCas9 known as nickase Cas9 (nCas9). The nCas9 only introduces single-strand breaks (nicks) in the DNA of the target region, owing to a mutation in its nuclease domain (H840A or D10A mutation in the HNH or RuvC1 domains, respectively) [35]. Therefore, even if nCas9/gRNA complexes bind to non-target sites and exhibit nuclease activity, only nicks are introduced. Because nicks can be accurately repaired using the unbroken DNA strand, sequence alteration as the off-target effects are unlikely to occur [36] (Figure 3a). The double-nicking method was developed by employing nCas9 and combining two different gRNAs [37] (Figure 3b). In this method, a pair of gRNAs are designed to flank the target sequence of the modifications. DSBs are induced only when two gRNAs are arrayed in a single genomic sequence. Thus, the gRNA recognition sequence is extended to approximately 40 bp, greatly reducing the probability of encountering similar sequences. The double-nicking method was further improved by replacing nCas9 with dead Cas9 (dCas9), which has mutations in both its nuclease domains and has lost its nuclease activity, fused with the FokI nuclease [38,39] (Figure 3c). Nicking can still cause off-target effects because nicks stall DNA replication forks and cause single-ended DSB [40]. The FokI nuclease only exhibits nuclease activity when it forms a dimer [38,39], and thus, it does not introduce a nick at off-target sites, and it more effectively suppresses off-target effects.

Although the double-nicking method effectively reduces off-target effects in non-target regions, it still breaks both strands of the DNA in the target region, which can result in unwanted mutations in the target regions when the donor DNA lacks sufficient nucleotide alteration in its target and PAM sequences (Figure 2). To address this limitation, the effectiveness of nCas9 alone for genome-wide base editing through HDR was demonstrated in yeast cells [33] (Figure 3d). In yeast, nicks do not promote NHEJ but promote HDR [41], owing to the ssDNA generated from nicks following the gap formation by 5′ exonuclease [42]. Therefore, the use of nCas9 for genome editing via HDR is expected to reduce unwanted mutations in the target sequence. A genome editing method was established by introducing a multi-copy plasmid containing the genes for the constant expression of nCas9 and gRNA as well as an approximately 1 kbp donor DNA sequence to provide a stable supply of donor DNA (CRISPR Nickase system) [33]. The CRISPR Nickase system was used to introduce nonsense mutations downstream of the gRNA target sequences, resulting in clones with no unwanted mutations but intended mutations. In contrast, when Cas9 was used, some clones with the intended mutation showed unwanted deletions or insertion mutations in the gRNA target sequences. Thus, only correct alterations were detected when using nCas9. Furthermore, insertion mutations could be accurately introduced 53 bp upstream and 50 bp downstream from the nick position without any unwanted mutations. These results indicate that the CRISPR Nickase system can be used for precise genome editing using donor DNA in a region corresponding to 97.2% of the yeast genome, expanding the effective range of precise genome editing in the yeast genome by 1.4-fold compared to the Cas9-based method.

Recently, engineering efforts focused not only on the mode of DNA strand breaks but also on considering the cleavage kinetics of Cas9 nuclease activity. To improve the kinetics for reducing off-target effects, a broad range of Cas9 variants was explored, and high-fidelity Cas9 variants were shown in mammalian cells; one of the Cas9 variants was validated in yeast [43]. The structural properties of the Cas9 protein, such as the interaction between the positively charged groove of Cas9 and the negatively charged phosphate group of the target DNA [44], the formation of an activated conformation for DNA cleavage following DNA binding of the gRNA/Cas9 complex [45], and domains that stabilize gRNA mismatches [26], play an important role in DNA cleavage activity. Therefore, by introducing mutations in these regions, the non-permissiveness of Cas9 nuclease activity to mismatches between gRNA and genomic DNA can be acquired. Rational modifications based on structural analysis and high-throughput screening were successfully employed to develop high-fidelity Cas9 variants. Some strategies were used for mutagenesis to reduce the cleavage of mismatched DNA strands. Mutations in the residues that contact the target DNA strand decrease the stabilization of binding to the non-target DNA strand [46,47]. The domains that form an intermediate inactive state for proofreading of the mismatched target DNA strands were also targeted [43,48], and whole residues of the Cas9 protein were screened using the directed evolution method [49]. However, these high-fidelity mutations also tend to disturb Cas9 cleavage kinetics at the correct target site, resulting in reduced on-target activity [50]. Recently, a loop that stabilizes mismatches located distally from the PAM sequence was characterized, and mutations in the loop were found to overcome the barrier of maintaining on-target activity during the construction of high-fidelity Cas9 mutants [26]. In the future, the integration of nicking strategies with high-fidelity Cas9 mutants could become a more effective method for reducing off-target effects [51].

**Figure 4:**
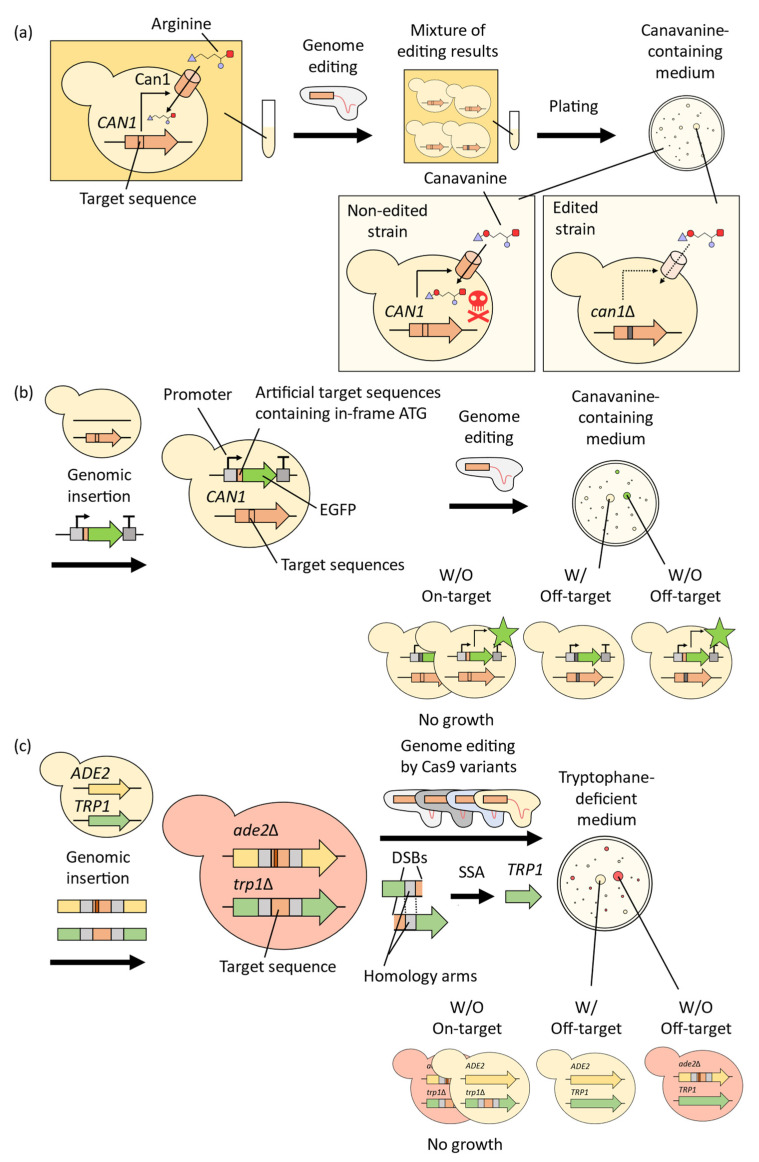
Confirmation of CRISPR-Cas9 results using yeast selective marker genes. (**a**) Results of genome editing targeting the *CAN1* gene, which encodes a transporter for the toxic compound canavanine, can be confirmed via growth selection in canavanine-containing medium. (**b**) EGFP sequence was inserted into the yeast genome after a promoter sequence and an artificial target sequence with an in-frame start codon. Following genome editing of the artificial target sequence, EGFP expression was disrupted, confirming the reduction in off-target effects, as evidenced by an increase in the number of cells showing GFP fluorescence. (**c**) Disruption of *ADE2* and *TRP1* by an inserted sequence flanked by homology arms can be repaired by SSA when DSBs are induced via genome editing. Yeast colonies with disrupted *ADE2* genes were red because of the accumulation of intermediate metabolites. Therefore, red-colored colonies grown in tryptophan-deficient medium expressed the high-fidelity Cas9 variants.

For the development of the yeast CRISPR-Cas9 system, efficient methods to evaluate the precision of genome editing are needed. The conspicuous cellular phenotypes of yeasts are useful for these methods due to readily available selective marker genes [3,33,43] (Figure 4). For example, the development of the CRISPR-Nickase system employed the conditional lethal gene *CAN1* to select strains in which genome editing inserted a stop codon into the sequence (Figure 4a). *CAN1* encodes an arginine transporter that can also uptake the toxic arginine analog (canavanine) in the medium [52]. Therefore, the occurrence of on-target genome editing on *CAN1* enables their growth in canavanine-containing medium. Sequencing the *CAN1* gene in colonies grown on canavanine-containing medium can efficiently evaluate unwanted mutations in target regions [33]. As a result, although CRISPR-Nickase inserted the stop codon into the target gene with 50% Cas9 use efficiency, the edited clones did not contain unwanted mutations. To validate off-target effects, the sequence of a fluorescent EGFP expression cassette was inserted into the yeast genome, and the artificial target sequence of gRNA was inserted downstream of the cassette’s promoter sequence (Figure 4b). The use of the CRISPR-Nickase system did not result in a reduction in the number of GFP-positive cells, indicating that off-target sites can be neglected even when the sequence in an off-target site is identical to the on-target site.

Yeast-selective markers have also been employed for high-throughput screening of high-fidelity Cas9 variants [43]. To validate off-target effects, nearly identical target sequences were inserted into the internal regions of the *TRP1* and *ADE2* genes. The target sequences were flanked by homology arms; thus, these gene sequences were restored via single-strand annealing (SSA) between these arms only when DSBs were induced at these sites [53] (Figure 4c). Thus, the yeast cells, in which the target site was cleaved by Cas9, only grow in tryptophan-deficient medium because the disruption of *TRP1* results in tryptophan auxotrophy. On the other hand, disrupting the *ADE2* gene leads to the formation of red-colored colonies, as the *ADE2* gene encodes an enzyme that converts a red-colored intermediate metabolite to a white one [54]. Therefore, when the gRNA recognizing the target sequence inserted in *TRP1* is expressed, only red colonies growing on tryptophan-deficient medium shows successful editing without off-target nuclease activity on the target sequence inserted in *ADE2*. As the opportunity for nuclease activity in *ADE2* increases with repeated strokes of the colonies, a low rate of white colonies with repeated strokes indicates more rigorous specificity of Cas9 variants [43]. Identified variants were additionally engineered and evaluated for their genome-wide off-target activity in mammalian cells using the GUIDE-seq method [10], which detects DSBs on a genome via specific double stranded oligodeoxynucleotides insertion. As a result, final high-fidelity Cas9 variants showed 79-fold improvement of specificity with 90% on-target activity [43]. These studies highlight the power of yeast genetics platforms to develop CRISPR-Cas9 systems with reduced off-target effects.

## 3. Application of CRISPR-Cas9 System to Epigenome Editing in Yeast

Epigenome editing was developed to overcome the limitations of the CRISPR-Cas9 system in modifying cellular processes independent of genome sequences. Possible candidates for the target of epigenome editing include chemical modifications of chromatin [13], transcriptional regulation by transcription factors [55,56], non-coding RNA associations with chromatin [57], and three-dimensional chromatin architecture [58]. Among these, the most well-defined targets for epigenome editing are chemical modifications of chromatin components, such as DNA methylation, nucleosomal histone acetylation, and methylation [13]. It is well-established that the epigenetic state affects chromatin condensation, the accessibility of transcription-related proteins, and gene expression levels. Therefore, changes in the epigenetic state are known to contribute to biologically significant phenotypes, such as cellular differentiation [59] and human diseases [60]. In *S*. *cerevisiae*, epigenetics is well-studied in transcriptional silencing at the *HM* locus that controls the mating-type switch, telomeres that establish chromosomal stability and nuclear spatial organization, and ribosomal DNA repeats that determine the length of the replicative lifespan [61]. In addition, certain epigenetically influenced phenotypes were reported, including the regulation of flocculation [62], respiratory functions of mitochondria [63], and UV hyper-resistance [64]. Furthermore, the physical accessibility of genome-editing enzymes to chromatin affects editing efficiency, which limits the potential for genome editing in both mammalian [65,66] and yeast cells [11,67,68]. Thus, advances in epigenome editing technology have become increasingly necessary as a method for reverse genetics-like research of epigenetics, a tool for the reversible regulation of gene expression in biotechnology, and a way to expand the functional capabilities of genome editing.

To achieve sequence-specific epigenome editing, enzymes related to epigenetics were combined with dCas9, a mutant Cas9 that lacks nuclease activity [69]. The conventional way of regulating the chromatin condensation state in eukaryotic cells uses chemical catalysts that react with histone proteins [70] or chemical inhibitors of histone modification enzymes [71,72,73]. Although these methods were effective in manipulating the global genomic epigenetic states, they lacked sequence specificity. In contrast, dCas9-fused enzymes are expected to be guided to the target sequence via gRNA recognition. This leads to site-specific epigenetic modifications through their action on the DNA and proteins in the surrounding area. Currently, alterations in gene expression at specific sites, which seem to be caused by changes in the chromatin state, have been achieved in mammalian cells. To induce loosened euchromatin states, histone acetyltransferase (HAT) [69], histone demethylase [74], and DNA demethylation enzyme [75] have been used. On the other hand, to induce condensed heterochromatin states [75], histone deacetylase [76] and DNA methyltransferases [77] have been used. In *S*. *cerevisiae*, genomic DNA contains almost no detectable levels of cytosine methylation [78,79,80]. Although some studies show the effects of DNA methylation on gene expression [81], editing the DNA methylation state for epigenome editing seems to be less meaningful because of the probable loss of native DNA methylation-related responses [82]. These fusion enzymes can specifically activate or repress the expression of target genes and have been used to establish CRISPR-mediated activation/repression systems in yeast cells [7,56,83]. However, these studies have often focused solely on measuring the transcriptional level or chromatin modification without thoroughly evaluating the changes in the chromatin condensation state [69,74,76]. Moreover, there have been no attempts to determine whether these tools can effectively achieve epigenetic modifications in genomic regions characterized by heterochromatin. Therefore, the development of epigenome editing systems capable of modulating the condensation state of chromatin in heterochromatic regions is necessary to perform broadly epigenetic studies in a more physiologically relevant context.

**Figure 5:**
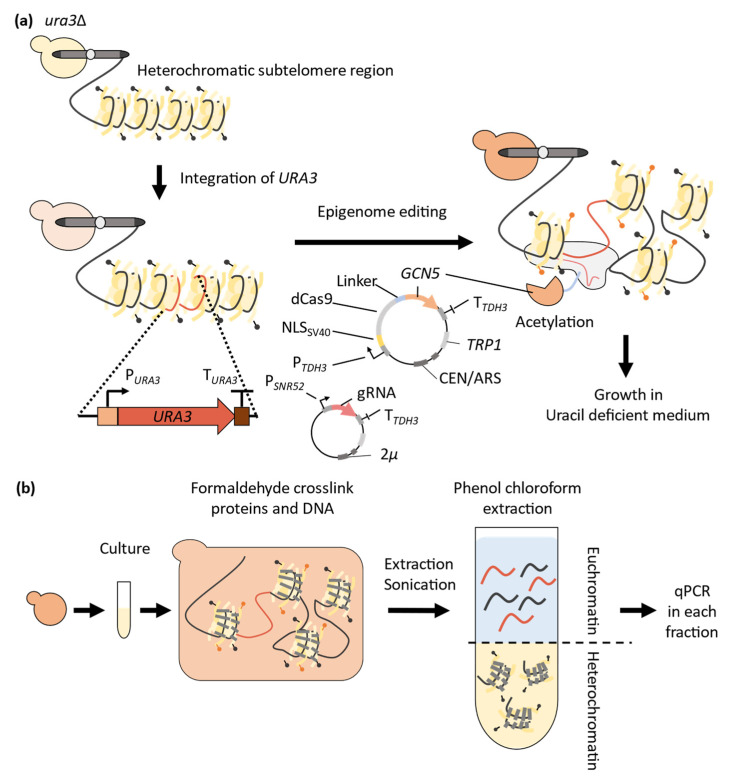
Epigenome editing in yeast for inducing chromatin loosening in the heterochromatic region. (**a**) The dCas9 protein fused with the Gcn5 histone acetyltransferase protein is expressed in yeasts with gRNAs that target the *URA3* gene in the subtelomere region. It is expected that Gcn5 is guided to the target region by the dCas9/gRNA complex and acetylates the histones in that region. (**b**) FAIRE-qPCR methods can quantify the chromatin condensation state of specific DNA sequences in the genome using qPCR after protein–DNA crosslinking and some preparation steps [84].

In a recent study, yeast cells were used to investigate the potential of the HAT-dCas9 fusion enzyme to shift a heterochromatin state to a euchromatin state [84] (Figure 5). The epigenome-editing enzyme was composed of the nuclear localization signal (NLS) of the SV40 large T antigen fused with the dCas9 and Gcn5 protein, which is a yeast HAT (Figure 5a). The fused enzyme (dCas9-Gcn5) was expressed on a centromeric-type plasmid with a strong constitutive *TDH3* (GPD) promoter. The fused enzyme was expressed within a uracil-auxotrophic yeast strain containing a complete native *URA3* gene expression cassette in the heterochromatin state of the subtelomere region [85], thereby improving growth in the absence of uracil and implying the potential for euchromatin induction in the heterochromatic *URA3* region. These findings indicate that the expression of dCas9-Gcn5 along with gRNA targeting the heterochromatic *URA3* region improved cell growth in uracil-deficient medium compared to the expression of dCas9-Gcn5 alone without gRNA [84]. Therefore, gRNA-dependent chromatin loosening in a heterochromatic region was suggested to be performed by dCas9-Gcn5 in a heterochromatic region.

Furthermore, euchromatic DNA was collected from yeast cells, and chromatic condensation levels were evaluated via optimized FAIRE-qPCR (Formaldehyde-Assisted Isolation of Regulatory Elements-quantitative Polymerase Chain Reaction) analysis to determine whether the euchromatin state was induced in the targeted heterochromatin region [86,87] (Figure 5b). FAIRE-qPCR provides a low-bias isolation of nucleosome-free DNA regions, avoiding immunoprecipitation and enzymatic reaction by using a formaldehyde DNA-protein cross-linking reaction. The study shows that the expression of dCas9-Gcn5 and gRNA induced the euchromatin state in the target region, and in the absence of gRNA expression, there were off-target inductions of the euchromatin state in other genomic regions [84]. Consequently, epigenome editing for the transition from the heterochromatin state to the euchromatin state in the targeted region was achieved by recruiting Gcn5 to the target region using gRNA.

The study also shows that the deletion of *ADA2* results in the loss of gRNA-dependent improvement in chromatin loosening [84]. Ada2 is an intracellular accessory protein that enhances the nucleosomal acetylation activity of Gcn5 [88]. This implies that the success of epigenome editing relies not only on the activity of dCas9-fused DNA- and histone-modifying enzymes but also on the contribution of multiple factors involved in the cellular epigenetic process. For better accuracy and reliability of epigenome editing, it is necessary to carefully examine and manipulate the influence of intrinsic intracellular factors associated with the modifying enzymes of epigenetic states. This issue could be especially important for therapeutic and breeding applications of epigenome editing, emphasizing the requirement for comprehensive engineering strategies that go beyond genome-editing enzymes alone.

## 4. Challenges to the Establishment of Mitochondrial CRISPR-Cas9 Systems in Yeast

Mitochondria are organelles that are found in numerous eukaryotic cells. They participate in various intracellular processes, including ATP synthesis via respiratory oxidative phosphorylation [89], the specific degradation and synthesis of various metabolites [90], and the regulation of apoptosis [91]. Electron transfer chain functions are also associated with the generation of harmful reactive chemical species such as reactive oxygen species (ROS) [92]. Therefore, mitochondrial dysfunction is frequently associated with adverse cellular processes such as cellular senescence [93]. Mitochondrial DNA (mtDNA) is one of the most distinctive features of these organelles. Many mitochondrial functions depend on gene products that are exclusively encoded in mtDNA [94], highlighting the importance of genome editing technology for mtDNA to completely edit the genetic information of a target cell. Moreover, mtDNA in mammalian cells harbors important genes whose mutations can result in human genetic mitochondrial diseases [15], including epilepsy [95] and dementia [96]. Thus, a system is necessary to alter the mtDNA sequence to the desired sequence, enabling the reverse genetic analysis of the vast unknown biological properties of mitochondria and the complete treatment of mtDNA-related diseases.

**Figure 6:**
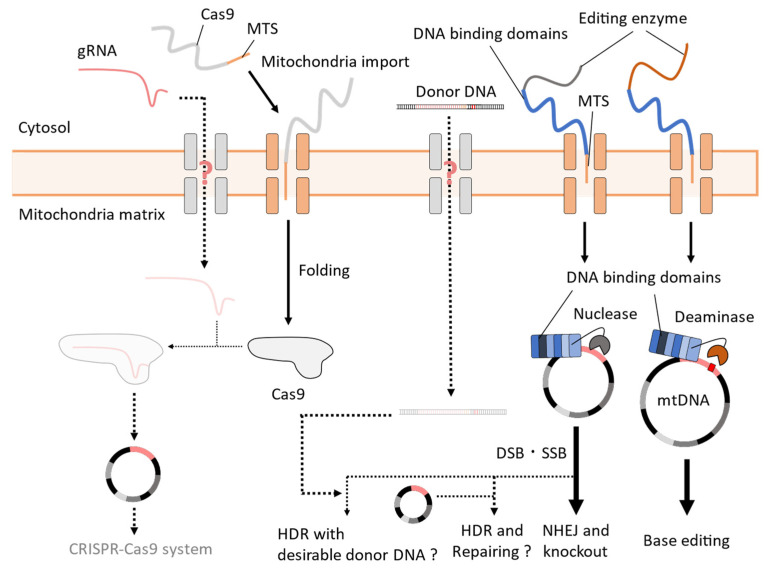
Difficulty in the mitochondrial transportation of elements necessary for mitochondrial genome editing. Any protein can be imported to the mitochondria by fusing with MTS. Thus, base editing can be performed using modular DNA-binding domains and deaminases. However, the native mitochondrial transportation of gRNA and donor DNA is unclear, making it difficult to introduce desirable sequences using the CRISPR-Cas9 system and HDR in mtDNA.

Despite these demands, the genome editing of mtDNA, particularly using the CRISPR-Cas9 system, remains highly challenging because of the difficulties in transporting gRNA and donor DNA into the mitochondria [21] (Figure 6). Although genome-editing enzymes such as Cas9 can be easily transported into the mitochondria via fusion with the mitochondrial target sequence (MTS) [19], it remains unclear how functional long polynucleotides, such as gRNA and donor DNA, can be transported to mtDNA. The use of intrinsic cellular mechanisms for this transport has not been well-established.

For the mitochondrial transportation of gRNA, the systems of using intracellular mechanisms such as the aforementioned improvements in the CRISPR-Cas9 systems have not been well-established. In mammalian cells, there have been controversial reports regarding the existence of sufficient mitochondrial RNA import activity for the CRISPR-Cas9 system [97]. The reduction of mtDNA by the expression of MTS-Cas9 and gRNA targeting the mtDNA sequence was reported in mammalian cells [98] and zebrafish [99]. Other studies show that the addition of specific sequences to gRNA, which is expected to form a secondary structure that facilitates mitochondrial transport, increases the localization of gRNAs in the mitochondrial matrix in mammalian cells [22,100,101]. However, these studies are insufficient to validate the induction of DSBs at the target sites on mtDNA and subsequent genome editing processes; therefore, the mitochondrial CRISPR-Cas9 system has not yet been established [21,96,102]. A recent report demonstrated the replacement of a 6 bp sequence in the mtDNA target site of gRNA by introducing exogenous single-stranded DNA oligonucleotides (ssODNs) with MTS-Cas9 and gRNA in mammalian cells [20]. Although this state-of-the-art result is attractive, future developments are required to enhance the editing efficiency and length of editable sequences. Therefore, MTS-fused modular DNA-binding proteins such as transcription activator-like effector nuclease (TALEN) and nucleic acid deaminase enzymes, which do not require gRNA and donor DNA for genome editing, respectively, have been fused as base-editing enzymes and transported into the mitochondria for genome editing [17,18,103]. However, this approach is apart from some benefits of the CRISPR-Cas9 system [19,20,21,104], including the ability to perform comprehensive reverse genetic analysis through the easy modification of gRNA target sequences [7] (Table 1). 

Furthermore, even if mtDNA modification occurs, the multi-copy nature of mtDNA in a cell makes it challenging to construct homozygous genotypes and alter cellular phenotypes. Additionally, the degradation of mtDNA resulting from DSBs is a barrier to mtDNA genome editing. Edited mtDNAs are degraded and replaced by wild-type mtDNA through its replication because mtDNAs are essential in mammalian cells; thus, edited mtDNA molecules in small fractions are rapidly depleted in cells [101]. Conventionally, a solution to this problem was attempted through the degradation of wild-type mtDNA by targeting its unedited sequence using MTS-fused ZFN or TALEN [105].

**Figure 7:**
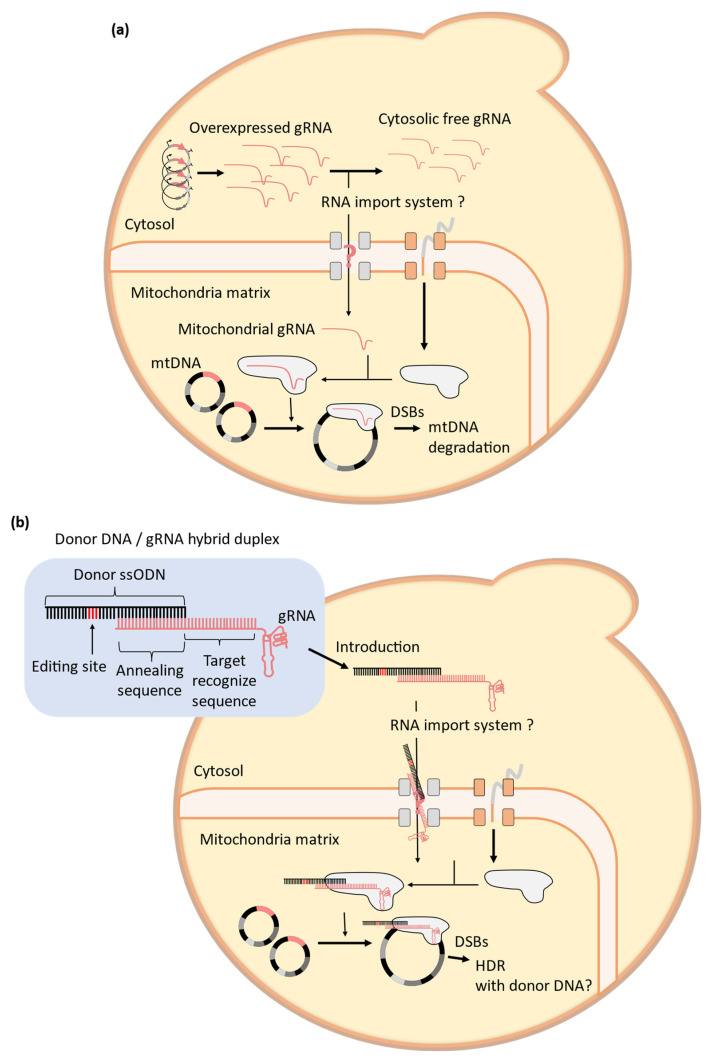
The potential of CRISPR-Cas9 systems targeting mitochondria in yeasts. (**a**) In yeasts, overexpression of mitochondrial targeting gRNA increases the rate of mtDNA degradation induced by the transport of Cas9 to mitochondria. Thus, the native mechanism of RNA transportation may also be applied to gRNAs in yeasts, and gRNA transportation could be used to recruit Cas9 to specific mtDNA regions and induce DSBs there. (**b**) The CRISPR-Cas9 system in yeasts can use a donor DNA / gRNA hybrid duplex, which may transport donor DNA components to mitochondria via the gRNA portion of the hybrid duplex.

*S*. *cerevisiae* can be used as an ideal model organism for investigating mtDNA dysfunction. In addition to its genetic tractability, yeast can grow via anaerobic fermentation without requiring any mitochondrial function. This feature makes it possible to study mtDNA genotypes that are lethal in other organisms [106]. Thus, yeast cultures under anaerobic fermentation conditions can avoid problems with mtDNA copy number maintenance [104]. A recent study demonstrated the potential of the CRISPR-Cas9 system to target mtDNA in yeast cells [104]. To efficiently construct cells lacking mtDNA (ρ °cells), Cas9 fused with MTS from *COX4* at the N-terminus (MTS-Cas9) was expressed on a centromeric plasmid with a strong constitutive *TDH3* (*GPD*) promoter. When gRNA targeting *ATP8* encoded by mtDNA was expressed on a multi-copy plasmid with the *SNR52* promoter, the formation rate of mitochondria-depleted cells that lost viability in non-fermentative glycerol medium was significantly increased in comparison to that of control cells without gRNA expression [104]. This result suggests that a certain portion of the expressed gRNA could enter the mitochondrial matrix in yeast cells through an intrinsic mechanism (Figure 7a). Therefore, yeast cells have the potential to perform mtDNA genome editing using the CRISPR-Cas9 system by simply expressing gRNA with a certain sequence [104]. The transport of nuclear-encoded tRNAs such as tRNA^Lys^_CUU_ [107] and tRNA^Gln^ [108] to the mitochondria was reported in yeast. It was reported that the fusion of their transport-related secondary structures with artificial RNAs enhances mitochondrial transport [109,110] and mtDNA depletion through the expression of MTS-Cas9 in mammalian cells [22]. Because it has been challenging to deliver gRNA to the mitochondria in mammalian cells [19], the ability to perform mitochondrial reverse genetics with the CRISPR-Cas9 system in yeast cells is expected to efficiently provide insights into mitochondrial cell biology, which can also be useful in understanding human mitochondrial diseases.

**Table 1:** A comparison of the latest targeted mitochondrial DNA-editing technologies in mammalian cells.

Editing Enzymes	DNA Targeting Elements	Types of Mutations	Editing Efficiency	Off-Target Editing Frequency of mtDNA	Refs
DdCBEs	TALE repeats	5′-TC-3′ to 5′-TT-3′	5–50%	0.024–0.049%	[111]
TALEDs	TALE repeats	A to G	49%	0.008–0.013%	[17]
HiFi-DdCBEs	TALE repeats	5′-TC-3′ to 5′-TT-3′	53–61%	0.001–0.0175%	[103]
MTS-Cas9	gRNA	Reduction of mtDNA	30–60%	Not reported	[22,98,100]
mito-Cas9 system	gRNA	Small sequence insertion	0.03–0.23%	Low	[20]

Conversely, there are no physiological mechanisms for the mitochondrial transportation of DNA molecules that were introduced into the cytosol for genome editing. However, yeast mitochondria possess a system of HR activated by DSBs [112]; thus, the easy method for the introduction of donor DNA molecules into the mitochondria facilitates more flexible editing of mtDNA [20]. To achieve physical transportation, membrane-permeable nanocarriers to transport donor DNA [113,114] and the particle gun method [23] for genome editing of mtDNA have been employed. Although promising methods have been shown, these techniques require additional alternative carrier materials with donor DNA, and a simpler method of donor DNA delivery is required. One potential method was suggested in recent studies on genome editing using DNA–RNA hybrid nucleic acids composed of donor DNA and gRNA [115] (Figure 7b). This method involves extending the 5′ ends of the target recognition sequence of gRNA pairing with 40 complementary bases at the 5′ end of a 121-base donor DNA to form a hybrid duplex. Electroporating this duplex into Cas9-expressing yeast cells was shown to effectively edit genome DNA with significantly higher efficiency than the separate introduction of donor DNA and gRNA [115]. It is currently unclear whether the hybrid duplex introduced into the cytosol is transported to the mitochondria through its gRNA portion, but this method suggests the potential of a novel easy delivery method of donor DNA to the mitochondria.

## 5. Summary and Future Perspectives

The CRISPR-Cas9 system is continuously advancing genome editing by developing technologies with various characteristics due to its ability to accept the fusion of other elements into Cas9 [116,117] and gRNA [118]. Recently, genome editing for medical applications in mammalian cells and breeding non-model organisms has received attention. However, efforts to establish novel genome-editing technologies using yeast cells, which are some of the simplest model organisms for genome modification, are still ongoing. Pursuing novel genome-editing technologies using yeast cells is still valid, as progress has been made in expanding the CRISPR-Cas9 system in yeast cells, including the reduction of off-target effects, epigenome editing, and mitochondrial genome editing. The use of the nicking activity of nCas9 is a core of emerging methods that have lower off-target effects, which have been successfully developed using yeast with its high HDR activity [33]. This concept was followed by more modern concepts of protein engineering, resulting in obtaining high-fidelity Cas9 variants through the use of the yeast platform with genetically tractable selective marker sets [43]. Beyond the DNA sequences, epigenome editing has focused on altering the chromatin chemical modification by fusing dCas9 to chromatin remodeling enzymes. Yeast has also been used in epigenome editing, such as in establishing a chromatin loosening system at a heterochromatic locus [84]. The most emerging topic is expanding CRISPR-Cas9 systems to mitochondrial genome editing, whereas the established successful example of modifying mtDNA sequences using Cas9/gRNA complexes remains insufficient in editing efficiency. In this area, yeast will be recognized as a possible organism for developing a mitochondrial CRISPR-Cas9 system suggested by mtDNA depletion by the MTS-Cas9 with normal gRNA expressions [104]. Yeast cells are excellent chassis for assessing and screening CRISPR-Cas9 systems for diverse applications.

In the future, improved CRISPR-Cas9 methods that address the current limitations of yeasts will be integrated into cutting-edge genome editing applications [5,6,7] and extend their capabilities to broader cellular processes. The proposed transport of RNA to mitochondria in yeast cells holds one of the most attractive aspects of yeast. Exploring the improvement of mtDNA editing by using powerful yeast genetic screening platforms would pave the way for the establishment of a mitochondrial CRISPR-Cas9 system. The emerging CRISPR-Cas9 system being developed in a user-friendly yeast cell format is likely to be extended to other organisms and will continue to be a driving force in the advancement of the entire field of genome editing.

## Data Availability

Data is contained within the article.
